# SpinWalk: A Monte Carlo simulator for MR-signal formation in inhomogeneous tissue

**DOI:** 10.1162/imag_a_00533

**Published:** 2025-04-15

**Authors:** Ali Aghaeifar, Sebastian Mueller, Klaus Scheffler

**Affiliations:** High-field Magnetic Resonance Center, Max Planck Institute for Biological Cybernetics, Tübingen, Germany; Department of Biomedical Magnetic Resonance, University of Tuebingen, Tübingen, Germany

**Keywords:** Monte-Carlo, diffusion, random-walk, microvascular, functional MRI simulation

## Abstract

Monte Carlo simulation is extensively utilized in functional magnetic resonance imaging (MRI) research to examine the behavior of an MR sequence in the presence of diffusion within complex microstructures. These simulations necessitate a substantial number of diffusing particles and time steps to be modeled to achieve convergence and produce robust and reliable results, which is computationally intensive. Incorporating additional parameters to enhance the realism of the simulations further intensifies this computational burden, particularly when simulating steady-state sequences, which require a long period of time to be simulated. To address this, we present SpinWalk, a high-performance Monte Carlo simulator for functional MRI. SpinWalk is free and open-source software, designed to offer a high-performance framework for facilitating the simulation of custom sequences. SpinWalk enables popular sequences in functional MRI to be efficiently simulated and ensures that results can be consistently reproduced. Key sequence and tissue parameters can be set, making SpinWalk flexible in examining different factors that contribute in signal formation. This versatility is demonstrated by replicating simulations from several previous studies, including GRE, SE, bSSFP, GRASE, and STE sequences. Performance evaluations demonstrate that SpinWalk can significantly reduce computation times, making it feasible to perform extensive simulations within a reasonable time frame.

## 1 Introduction

A Monte Carlo simulation is a computational technique employed to simulate the probability of various outcomes in complex systems or processes where predictions are challenging due to the involvement of random variables, or the absence of analytical models. Monte Carlo simulation has been widely applied in the field of magnetic resonance imaging (MRI) across various applications (e.g.,Baxter & Frank, 2013;Garpebring et al., 2013;Harkins et al., 2021;Prange & Song, 2009;Soher et al., 2001;Storey & Novikov, 2024). Particularly noteworthy is its application in modeling functional MRI contrast (Báez-Yánez et al., 2017;Khajehim & Moghaddam, 2017;Martindale et al., 2008) and investigating diffusion phenomena within microstructure (Lee et al., 2021;Rafael-Patino et al., 2020;Yeh et al., 2013), where Monte Carlo simulation has garnered significant interest since the complexity of the systems prevents the derivation of analytical solutions to generate ground-truth data.

Monte Carlo approaches allow inclusion of various biological characteristics and configurations, such as microvascular networks (Báez-Yánez et al., 2017), intravascular magnetic susceptibility (Boxerman et al., 1995), transverse relaxation (Weisskoff et al., 1994), mixture of axonal radii (Lee et al., 2020), or cell membrane permeability (Yeh et al., 2013) into the simulation. However, achieving stable and reliable simulation results requires a sufficiently large number of samples both for spins as random walkers and time steps (Fieremans & Lee, 2018). Also, an accurate representation of the diffusion media’s geometry and the physical properties of the underlying biological tissue or structure are crucial. Therefore, it is important to carefully consider available computation power and optimize the Monte Carlo simulation program accordingly. To mitigate computational burden, simplifications are often implemented, including simple measures such as using a smaller number of random walkers and time steps, a limited sample size, simple microgeometry, or more sophisticated approaches like confining 3D diffusion to 2D scenarios.

When subjected to magnetic field inhomogeneity, such as that found in BOLD fMRI, spins (here the protons of water molecules) experience off-resonance and accumulate phase, a process known as dephasing. The local field inhomogeneity, caused by susceptibility differences between blood and surrounding tissue, varies with the oxygenation level of blood. Moreover, spins may diffuse in all directions within the imaging voxel, experiencing different local field perturbations over time. Consequently, the dephasing rate can vary spatially and temporally. As the formation of MR signal includes the vector sum of all individual water proton magnetization within the imaging voxel, each with Brownian microscopic motion and a certain accumulated phase, signal attenuation occurs. This effect can also be observed in non-BOLD fMRI studies. For instance, recent work has explored how susceptibility-induced internal gradients within the extra-axonal and myelin compartments influence the pulsed gradient spin-echo (PGSE) signal (Winther et al., 2024).

Studies have evaluated various MR sequences in terms of their sensitivity to intravascular magnetic susceptibility changes in microvascular networks. These investigations have demonstrated strong agreement between Monte Carlo simulations and experimental findings. Specifically, the relationship between BOLD sensitivity and/or vessel size and MR signal contrast has been explored in gradient echo and spin echo sequences (Báez-Yánez et al., 2017;Boxerman et al., 1995), as well as in steady-state sequences such as bSSFP (Báez-Yánez et al., 2017;Bieri & Scheffler, 2007), FISP, and PSIF (Khajehim & Moghaddam, 2017;Scheffler et al., 2019), and CPMG and GRASE echo trains (Scheffler et al., 2021).

Robust and reliable Monte Carlo simulations require using a very high number of spins and time steps; otherwise, convergence may not be achieved (Hall & Alexander, 2009). Especially in so-called steady-state sequences involving preparation phases, the number of time steps that need to be simulated significantly increases compared to, for instance, a computationally rather simple GRE sequence. This challenge is further stressed when simulating substrates with long$T_{1}$values, as the preparation steps become longer. For instance, as a rule of thumb, the time for a bSSFP sequence to reach the steady state is approximately five times the$T_{1}$value (Bieri & Scheffler, 2013). Achieving a steady state for gray matter with a$T_{1}$value in the range of 2 s at ultrahigh-field MRI requires over 1000 RF pulses (withTR<10 ms). This emphasizes why a high-performance simulation tool is required to be able to lift restrictions on the type of sequence that can be investigated. Monte Carlo simulation of such scenarios is computationally expensive, and depending on the number of spins and available computing power, programs written in commonly used prototyping scientific languages like MATLAB and Python can take from several hours to a day to complete execution. If the contribution of a group of parameters such as blood volume, oxygenation level, orFAandTRneeds to be evaluated separately, the simulation needs to be repeated from the beginning, which can extend over several days or even a week in total. Therefore, there is a need for a versatile framework that can execute such simulations optimally within a reasonable time frame.

Several open-source, community-developed tools exist for performing Monte Carlo simulations in the context of diffusion MRI. Notable examples include MC/DC (Rafael-Patino et al., 2020), MCMRSimulator (Cottaar, 2023), Camino (Cook et al., 2006), Disimpy (Kerkelä et al., 2020), RMS (Lee et al., 2021), MATI (Xu et al., 2024), and simDRIFT (Blum & Utt, 2023). It is worth mentioning the matrix formalism method (P. T. Callaghan, 1997) as a computationally efficient alternative to the Monte Carlo approach for diffusion simulations in well-known geometrical models. MISST (Ianuş et al., 2016) is a widely used simulator of this type. While these tools offer advanced features for simulating various aspects of diffusion, they may not be fully equipped for simulations of BOLD fMRI. Some of the limitations are: 1) simulating only DWI sequences with diffusion sensitization gradients, 2) simplifying by excluding off-resonance effects and/or tissue relaxations, 3) inability to incorporate microvascular networks into simulations, and 4) computational speed constraints that limit their usability for a wide range of sequences.

Below, we present our implementation of the Monte Carlo simulator, SpinWalk. SpinWalk is a high-performance simulator developed in C++ and harnessing CUDA technology to leverage GPU acceleration. In the absence of a GPU device, SpinWalk can seamlessly switch to a CPU-only mode. The core simulation routines are designed to exploit massively parallel processing capabilities. While this work primarily focuses on applying SpinWalk to BOLD contrast in functional MRI, and it is the main motivation to develop this software, SpinWalk is not confined to this application. It serves as a versatile framework for conducting realistic, rapid, and precise Monte Carlo simulations of spin diffusion. As such, it can be utilized for Monte Carlo simulation across various aspects of diffusion MRI too. It not only enables simulation of DW-SE sequences with PGSE (Stejskal & Tanner, 1965) or oscillating gradient spin-echo (OGSE) (Does et al., 2003) settings, but also allows DW-SSFP simulations (McNab & Miller, 2010).Table 1provides a comparison between the aforementioned simulators and SpinWalk. To the best of our knowledge, there are currently no publicly available simulators specifically designed for performing Monte Carlo simulations of BOLD fMRI. The publicly available projects we identified are primarily focused on simulating diffusion-weighted MR signals using diffusion encoding gradients, typical of PGSE experiments. Except for MCMRSimulator (Cottaar, 2023), effects such as off-resonance, relaxation, and RF excitation are excluded for simplicity, limiting flexibility to arbitrary gradient waveforms. SpinWalk allows for an arbitrary number of substrates within the phantom, each with distinct relaxation constants, diffusivity, and permeability, and supports an arbitrary number of RFs and gradients with various shapes.

**Table 1.** List of some of open-source simulators for diffusion MRI, including their programming languages, licenses, supported computing units, and key features.

Project name	Programming language	License	CPU/GPU	Features	Reference ^1^
MC/DC	C++	LGPL-2.1	CPU	Arbitrary gradients, save trajectory, triangle mesh phantom	2c2ed25
Disimpy	Python	MIT	GPU	Arbitrary gradients, save trajectory, triangle mesh phantom	948c439
MCMRSimulator	Julia	Apache 2.0	CPU	Arbitrary gradients & RF, relaxation, off-resonance, membrane permeability, thorough documentation	1eab9ef6
MISST	MATLAB	Artistic 2.0	CPU	Arbitrary gradients	0.95
simDRIFT	Python	BSD-3-Clause	GPU	Gradients, NIfTI I/O, save trajectory	15756bb
SpinWalk	C++	MIT	CPU & GPU	Arbitrary gradients & RF, multi-echo, relaxation, off-resonance, save trajectory, HDF5 I/O, voxel mesh phantom, permeability	1.15

The listed features for each simulator is derived from information available in their repositories, manuals, or the original publications introducing each simulator.

1 Commit ID, released data, or released version.

SpinWalk is designed to use a predefined numerical phantom. Phantoms with simple structures such as parallel cylinders or spheres can be generated with SpinWalk. Phantoms with more complex structure may be generated with tools like MEDUSA (Ginsburger et al., 2019), CACTUS (Villarreal-Haro et al., 2023), ConFiG (R. Callaghan et al., 2020), etc. The process of determining the MR signal can be divided into two main stages: first, the creation of a phantom that represents the microstructural geometry and the corresponding off-resonance maps; and second, the execution of the random walk simulations based on the provided phantom. SpinWalk focuses on performing the random walk and simulating MR sequences but not on creating a phantom. Apart from the aforementioned toy examples, it is assumed that the microstructures and off-resonance maps are pre-generated and provided as three-dimensional matrices, which serve as input for SpinWalk. In this sense, any sophisticated model may be integrated into SpinWalk. If a complex microvascular model incorporating network dynamics, such as those proposed in Báez-Yánez et al. (2023) andHartung et al. (2021), is available, SpinWalk can be used to produceΔCBF,ΔCBV, andΔSO2 contrast—of course dependent on what was implemented in the model—and distinguish different signals within a laminar profile. SpinWalk is open source, and we encourage contributions from the community via the project’s GitHub repository athttps://github.com/aghaeifar/spinwalk.

## 2 Theory

### 2.1 Phase evolution during random walk

Monte Carlo simulation in the context of BOLD fMRI can be explained as phase accumulation of spins (acting as random walkers) diffusing within a medium with a non-uniform magnetic field. The accumulated phase for the$s^{th}$spin in each diffusion time step,dt, can be stated as:

$$\Delta \Phi_{s}\left(t\right)=\gamma \Delta B_{z}\left(\vec{r},t\right)dt$$ (1)

whereγis the gyromagnetic ratio, and$\Delta B_{z}$represents the local field deviation. The MR signal is then formed from the vector sum of spins that are out-of-phase because of experiencing different precessions. The amplitude of signal at the echo time,$S_{TE}$, after an exemplary 90° RF excitation can be expressed as:

$$S_{TE}=\sum_{s=1}^{Ns}e^{i\Phi_{s}}e^{-TE/T_{2,s}}$$ (2)

where

$$\Phi_{s}=\sum_{t=1}^{Nt}\Delta \Phi_{s}\left(t\right)$$ (3)

andNsis number of spins,Ntis number of time steps, equal toTE​/​dt, and$T_{2,s}$indicates the transverse relaxation for$s^{th}$spin. Any gradients or refocusing RF pulses occurring between excitation and echo-time should be incorporated into$\Phi_{s}$. Transverse relaxation,$T_{2}$, arising from intrinsic spin-spin interactions, is combined with dephasing caused by local field inhomogeneities and diffusion, leading to$T_{2}^{*}$relaxation, and is already inherent in$S_{TE}$. The local magnetic field perturbation plays a vital role in BOLD fMRI Monte Carlo simulations and is the origin of the contrast, whereas it can typically be ignored in simulations of diffusion MRI since the signal contribution due to the BOLD effect is comparatively small. The local field inhomogeneity originates from susceptibility differences between the tissue and paramagnetic deoxyhemoglobin in the blood vessels or other structures. Reduced oxygenation level increases this susceptibility difference, leading to a stronger signal attenuation.

The expected mean displacement for each time stepdtis determined by Einstein’s equation:$\langle r\rangle =\sqrt{2N_{d}Ddt}$, where$N_{d}$represents the spatial dimension andDis the diffusion coefficient. This displacement can be understood as the standard deviation of a Gaussian distribution with a mean of zero. In practical terms, diffusion can be simulated by combining three 1D random walks along orthogonal axes in a Cartesian coordinate system ($r=\sqrt{2Ddt}$) or by a single 3D random walk in spherical coordinates ($r=\sqrt{6Ddt}$) and randomly choosing azimuthal and polar angles. Practically, the position of the$s^{th}$particle at time$t_{n+1}=t_{n}+dt$is updated as

$$r_{i,n+1}=r_{i,n}+dr_{i}$$ (4)

withi=x,y,zanddrdrawn from a Gaussian distribution with mean zero and standard deviation$\sqrt{2Ddt}$.

### 2.2 Model of vasculature and physiology

The focus of SpinWalk lies on efficient calculations of a random walk and the simulation of MR sequences. The underlying field inhomogeneities may in simple cases, such as for instance in the case of parallel cylinders, be determined with SpinWalk, otherwise the field inhomogeneities are assumed to be provided from external sources as an HDF5 file (see the detailed implementation of the simulator below).

Typically, simulations are repeated multiple times with different local magnetic field inhomogeneities mimicing physiological response. Repeating the simulation twice is probably most common, for example, assuming instantaneous changes in blood oxygenation and volume in all vessels between rest and active states of fMRI examinations. However, for a more detailed tracking of signal evolution, the Monte Carlo simulation can be repeated throughout the entire course of the haemodynamic response function (HRF). To do so, a biophysical model is desired (e.g.,Báez-Yáñez et al., 2023;Hartung et al., 2022;Karch et al., 1999;Linninger et al., 2019) which allows the prediction of blood flow, volume, and oxygenation with finer temporal resolution and accordingly predicts the evolution of field inhomogeneities.

Many previous studies have used infinite cylindrical (Báez-Yánez et al., 2017;Khajehim & Moghaddam, 2017) or spherical (Bieri & Scheffler, 2007;Scheffler et al., 2019;Weisskoff et al., 1994) perturbation models to represent vessels and blood cells. These models are not only flexible enough to reflect the effects of parameters such as blood volume, size, and orientation to the main magnetic field, but they also provide an analytical solution for the field perturbation. For more realistic models, yielding more complex geometries for which no analytical solution can be provided, the off-resonance map can be calculated by convolution of a dipole kernel with a spatial mask of susceptibilities as demonstrated byPathak et al. (2008)andGagnon et al. (2015).

## 3 Simulator Implementation

### 3.1 Programming

SpinWalk is a command line tool, developed entirely in C++ to ensure efficient resource usage and high performance in computationally intensive tasks and memory management. The project leverages CUDA technology for parallelization of computations. Although it significantly increases computation time, if necessary due to the absence of a GPU, the computation can be configured to run entirely on the CPU. Additionally, the simulator utilizes the Boost and HDF5 libraries, both of which are open source and compatible with widely used platforms. Parallelization on the CPU utilizes the Thread Building Blocks (TBB) library, which has been part of the C++ standard since C++17. This makes the project highly portable across different operating systems, enabling it to run in diverse environments. During execution, spins are distributed across GPU (or CPU) cores to undergo the diffusion process while also experiencing the simulated effects of a certain MRI sequence.

### 3.2 Framework overview

A detailed overview of SpinWalk, along with practical examples and a detailed documentation, is available on the SpinWalk GitHub repository. This section provides a concise introduction to the key concepts. The initialization of the simulation is specified through one or multiple text-based configuration files using the INI structure and syntax. Each configuration file represents an individual simulation and is passed as an argument to the simulator. The configuration file includes all the parameters required to define the simulation and can be adjusted by the user. These parameters encompass sequence definition, properties of the substrates such as relaxation times T1 and T2, input/output (I/O) files, and general settings. If multiple configurations (e.g., to assess the impact of TE) and several GPUs are available, such as in HPC clusters, each configuration can be assigned to a separate GPU or be executed sequentially on a single GPU.

#### 3.2.1 Sequence

Users have the possibility to configure RF pulses, TE, repetition time (TR), and gradients (across three axes) within the simulator. The simulator accommodates multiple RF pulses per TR, each with its own unique flip angle, phase, and onset. Moreover, multi-echo acquisition is supported. Custom gradient shapes for each axis can be generated from provided samples. Dephasing can be achieved using either gradients or an ideal dephasing mechanism (i.e., manually distributing phase). To establish steady-state conditions, users can specify the number of preparation pulses and define a certain phase-cycling. The dwell time for the RF pulse corresponds to a single diffusion time step. Accordingly, an RF waveform is applied as a sequence of hard pulses, occurring instantaneously and separated by intervals equal to one diffusion time step.

#### 3.2.2 I/O files

All input and output files are structured using the Hierarchical Data Format 5 (HDF5), renowned for its versatility and efficiency in data storage. It can be easily read and written in widely-used programming languages such as MATLAB and Python. The only required input file is the so-called “phantom” (VAN, cylinders, etc.), defining substrate boundaries and corresponding off-resonance maps if desired for inclusion in simulation. Multiple phantom files can be specified in one configuration file, allowing for identical sequence simulations across all. Optionally, users can provide initial magnetization and spin placement through additional input files. Otherwise, spins at equilibrium (i.e.,$M0_{xyz}=\left[0,0,1\right]$) will be randomly distributed among substrates. Simulations of each phantom file produce a single output file containing all relevant variables, such as spins’ magnetization at TEs and trajectory of random walks.

#### 3.2.3 Substrates

The geometry and domains of substrates are defined in a discrete voxel space, referred to in this work as a*voxel mesh*, and are stored as a 3D array in the phantom file. This array consists of integer values, each corresponding to a specific substrate. Each substrate has its own relaxation parameters ($T_{1}$and$T_{2}$) and diffusivity, specified in the configuration file. The movement of a spin between substrates during a random walk is controlled by a probability matrix of sizeN×N, whereNis the number of substrates. In this matrix,$P_{ij}$at the$i^{th}$row and$j^{th}$column represents the probability of permeation from substrateito substratej. A value of$P_{ij}=0$indicates an impermeable boundary. A fixed probability method for modeling permeability has also been previously utilized in a Monte Carlo simulation of skeletal muscle tissue structure (Hall & Clark, 2017). In this work, if a spin cannot move between substrates due to permeability restrictions, a new random step will be drawn in case; otherwise, a boundary would have been crossed with the next step. For very large time scales, local variations of diffusivity will ultimately lead to an accumulation of particles in areas with lower diffusion. This can be seen as a limitation of the simulation, which, of course, can never represent all features of the real world, but the effect of this might at least be directly investigated using SpinWalk by, for example, comparing uniform against spatially depended diffusion. Alongside the substrate voxel mesh, a 3D array of the off-resonance map with the same dimensions can be stored. This off-resonance map results from susceptibility differences between substrates, and its absolute value scales with the static magnetic field. For convenience, this map should be calculated for$B_{0}=1T$and will be internally scaled by the simulator.

#### 3.2.4 General settings

General simulation parameters, including the number of spins and the static field strength, can be specified. Also, different spatial boundary conditions are implemented: if a spin exits the region of space occupied by the substrate, it can either re-enter from the opposite side, creating a periodic simulation environment, or be reflected back to remain within the volume. None of these boundary conditions might fully reflect the reality but the presented framework allows for a straightforward comparison of their effects on the resulting signal.

## 4 Methods

The performance and capabilities of SpinWalk are demonstrated by reproducing results from previous works (Báez-Yánez et al., 2017;Bieri & Scheffler, 2007;Boxerman et al., 1995;Kim et al., 2012;Scheffler et al., 2019,^2021^;Uludağ et al., 2009). All simulations were conducted on a desktop PC equipped with an AMD Ryzen Threadripper PRO 5995WX CPU (2.70 GHz base frequency, 64 cores, 2 threads per core) and an NVIDIA RTX 6000 GPU (compute capability 8.9, 48 GB GDDR6 DRAM), unless stated otherwise.

### 4.1 Cylinder model

The utilization of cylinder-based models to represent vessels is widespread due to their adaptable nature, facilitating exploration of parameters such as volume fraction, vessel radius, and their alignment relative to$B_{0}$. Furthermore, this approach offers an analytical solution for determining the resulting magnetic field inhomogeneities, making it especially suitable for simulating BOLD responses. The field inhomogeneities induced by an infinite cylinder can be expressed as (Ogawa et al., 1993):

$$\Delta B_{z}\left(r\right)=\{\begin{array}{cc} 2\pi B_{0}\left(1-Y\right)\Delta \chi {\left(\frac{R}{r}\right)}^{2}\text{cos}\left(2\phi \right){\text{sin}}^{2}\theta & r\geq R\\ 2\pi B_{0}\left(1-Y\right)\Delta \chi \left({\text{cos}}^{2}\theta -\frac{1}{3}\right) & r<R \end{array}$$ (5)

In the equation,$\Delta B_{z}$represents the change in the magnetic field relative to$B_{0}$, whileΔχdenotes the susceptibility difference between fully deoxygenated blood and tissue. The variableYcontrols the blood oxygenation level, ranging from 0 to 1, with 1 indicating fully oxygenated. Additionally,Rstands for the radius of the vessel,rcorresponds to the Euclidean distance from the spin location to the axis of the cylinder,ϕrepresents the angle betweenrand the projection of$B_{0}$onto the plane orthogonal to the cylinder axis, andθsignifies the angle between the cylinder axis and$B_{0}$. Given the FoV and grid size, SpinWalk can create a phantom in which a specified fraction of the volume is occupied by randomly positioned, infinitely long cylinders of a certain radius and compute the corresponding field map according toequation (5). Note that modeling intravascular field inhomogeneities withequation (5)may not be fully accurate due to the dynamic nature of blood cells, which act as sources of strong local dipolar fields. These cells are in constant motion, changing shape and exchanging water molecules (Gagnon et al., 2016). However, for the purposes of demonstration and to maintain consistency with the studies being reproduced,equation (5)is still employed to estimate intravascular field inhomogeneities in this work.

### 4.2 Virtual FoV scaling

In the case of spatially isotropic oriented cylinders, for example, all randomly or all parallel to each other, the evaluation of different vessel sizes can be efficiently conducted using a single numerical phantom. This approach eliminates the need to generate multiple phantoms and avoids the necessity to determine different fieldoffset maps by scaling the FoV and voxel resolution appropriately. For instance, a phantom with an FoV of 500 µm and a grid size of 1000 (equals a voxel mesh resolution of 0.5 µm), containing a cylinder with a radius of 10 µm, can be alternatively used as a phantom with an FoV of 1000 µm with a voxel mesh resolution of 1.0 µm, containing a cylinder with a radius of 20 µm. The scaling is achived by modifying step width in the diffusion process such that the cylinders appear to be smaller or bigger compared to the original step size. The fieldmap calculated usingequation (5)for the original phantom is applicable to the scaled phantom. This methodology avoids creation of numerous environments containing different vessel sizes and maintains a consistent volume fraction across simulations with different vessel sizes. SpinWalk is capable of processing an array of FoV scaling factors given in the configuration file and executing identical Monte Carlo simulations for a single numerical phantom across various FoV scalings.

To evaluate the accuracy and stability of the FoV scaling approach, numerical phantoms with an FoV of 1000×1000×1000 µm^3^and a grid size of 1000 in each dimension were generated, each containing cylinders with a volume fraction of 4%. The cylinder radii were chosen from the set [1, 2, 5, 8, 12, 20, 35, 50, 75] µm. For each cylinder radius, 10 phantoms with randomly positioned cylinders were created, resulting in a total of 90 numerical phantoms. Signal change using the GRE sequence, as described in subsection*Simulations*, was simulated in each phantom while it was virtually scaled to mimic a phantom with a cylinder radius of 8 µm. The mean and confidence intervals of 95% (= mean±1.96×std /$\sqrt{\text{10}}$) for each radius were calculated as criteria for assessment.

The relationship between the original phantom’s FoV and cylinder size with virtual FoV scaling was analyzed by repeating experiments on phantoms with FoVs of 2000 µm and 4000 µm, each containing cylinders with a radius of 75 µm. Additionally, the impact of voxel mesh resolution was assessed using a phantom with a 500 µm FoV filled with cylinders of 1 µm radius. This was done by creating phantoms with grid sizes of [250, 500, 1000, 2000], corresponding to voxel mesh resolutions of [2, 1, 0.5, 0.25] µm.

### 4.3 Simulations

Phantoms with an FoV of 600×600×600 µm^3^and a grid size of 1200 were generated for sequence simulation. They were filled with cylinders of 8 µm radius, and virtual FoV scaling was applied when simulating different vessel sizes. BOLD sensitivity was assessed as a function of vessel size, vessel orientation, and blood volume fraction (BVF). For vessel size and orientation examinations, the volume fraction of cylinders was set to 4%; for BVF assessments, it ranged from 1% to 6%. All cylinders were aligned perpendicular to the B_0_direction, except in the vessel orientation assessment, where the angle between the cylinders and B_0_varied linearly from 0°to 90°.

Unless otherwise stated, all simulations with cylinder models were performed using the following parameters: B_0_= 9.4 T, T_1_[IV, EV] = [2500, 2200] ms; T_2_[IV_Y = 78%_, IV_Y = 85%_, EV] = [13, 20, 41] ms (Khajehim & Moghaddam, 2017); oxygenation levelY[rest, active] = [78, 85]%;Δχ= 0.11 ppm in cgs units; number of spins = 5×10^5^; time step = 50 µs; diffusion constant [IV, EV] = 1.0 µm^2^/ms, and impermeable surfaces. Additionally, a simulation was conducted in a phantom with a BVF of 4%, featuring permeable surfaces that allow water to cross vessel boundaries into tissue with a 10% probability. Spins were constrained to remain within the FoV; if they attempted to exit, their direction of movement was reversed to keep them inside.

The evaluations were conducted using the following sequences and changes in signal percentages (1 - S_rest_/S_active_) or signal differences (S_active_- S_rest_) were computed for extravascular contributions:

- Gradient Recalled Echo (GRE): TE = 20 ms.
- Spin Echo (SE): TE = 30 ms.
- Balanced Steady State Free Precession (bSSFP): TE/TR = 5/10 ms, FA = 20°, phase cycling = 180°, number of preparation pulse = 1100.
- GRAdient and Spin Echo (GRASE) (Constable et al., 1994;Feinberg & Oshio, 1991): nine spin-echos with 40 ms echo spacing and one gradient-echo in between each,$90_{x}^{\circ }$excitation RF followed by train of perfect$180_{\pm y}^{\circ }$refocusing RF.
- STimulated Echo (STE): a$90_{x}^{\circ }$excitation RF pulse was followed by two$90_{y}^{\circ }$refocusing RF pulses. The initial refocusing RF pulse was applied at 20 ms, and the mixing time (T_m_, the duration between the refocusing pulses) was individually assessed at 40, 80, 200, 500, and 1000 ms.

To examine the impact of varying diffusivity and permeability on particle density, additional simulations were conducted using a phantom composed of two substrates, each occupying 50% of the volume. In these simulations, 5×10^4^spins were randomly distributed throughout the phantom and allowed to diffuse for 1 s, with the diffusion trajectory being recorded over the entire simulation period. Three scenarios were examined: 1) both substrates had identical diffusivities, and the membrane between them was bidirectionally permeable; 2) the diffusivity of substrate 1 was twice that of substrate 2, while the membrane remained bidirectionally permeable; and 3) the diffusivities were identical, but particles could only diffuse from substrate 1 to substrate 2 with a 10% probability (unidirectional permeability), while diffusion in the reverse direction was prohibited.

### 4.4 Performance benchmarking

The total number of operations is proportional toU=NT, whereNis the number of walkers andTis the number of time points (Hall & Alexander, 2009). Given the architecture of SpinWalk, which utilizes a voxel mesh model to represent the numerical phantom, the FoV and grid size are additional factors that can influence computation time. To evaluate the impact of these factors, three phantoms with the following FoV and grid size combinations were generated: [1800 µm and 600], [600 µm and 600], and [600 µm and 1200]. The phantoms were filled with cylinders having a radius of 8 µm and a total volume fraction of 4%. Off-resonance was calculated according to equation(5)and incorporated into the simulation together with relaxation effects. For each phantom, 10^5^, 4×10^5^, and 10^6^spins were randomly distributed, and 10^4^random walks were performed in separate runs. The simulations were executed separately on both GPU and CPU, and the elapsed time was recorded.

With regards to performance benchmarking, a comparison between SpinWalk, simDrift (Blum & Utt, 2023), and Disimpy (Kerkelä et al., 2020) was conducted for a free diffusion experiment where 10^6^spins performed 10^4^random walks. Off-resonance and relaxation effects were excluded in SpinWalk, as these inputs are not considered in simDrift and Disimpy. All three simulators ran on a GPU, and simulations were performed for 2 and 100 different b-values, with the elapsed time recorded. To minimize unnecessary overhead, Disimpy simulations were run in quiet mode, and the simDrift source code was modified to suppress progress display. Disimpy and simDrift employed bipolar diffusion-sensitizing gradients (GRE-based sequence) in the absence of an RF pulse, with the magnetization already flipped. In contrast, SpinWalk used monopolar diffusion-sensitizing gradients (SE-based sequence), incorporating excitation and refocusing pulses, each lasting one time step.

## 5 Results

### 5.1 BOLD fMRI simulations

**Fig. 1.**
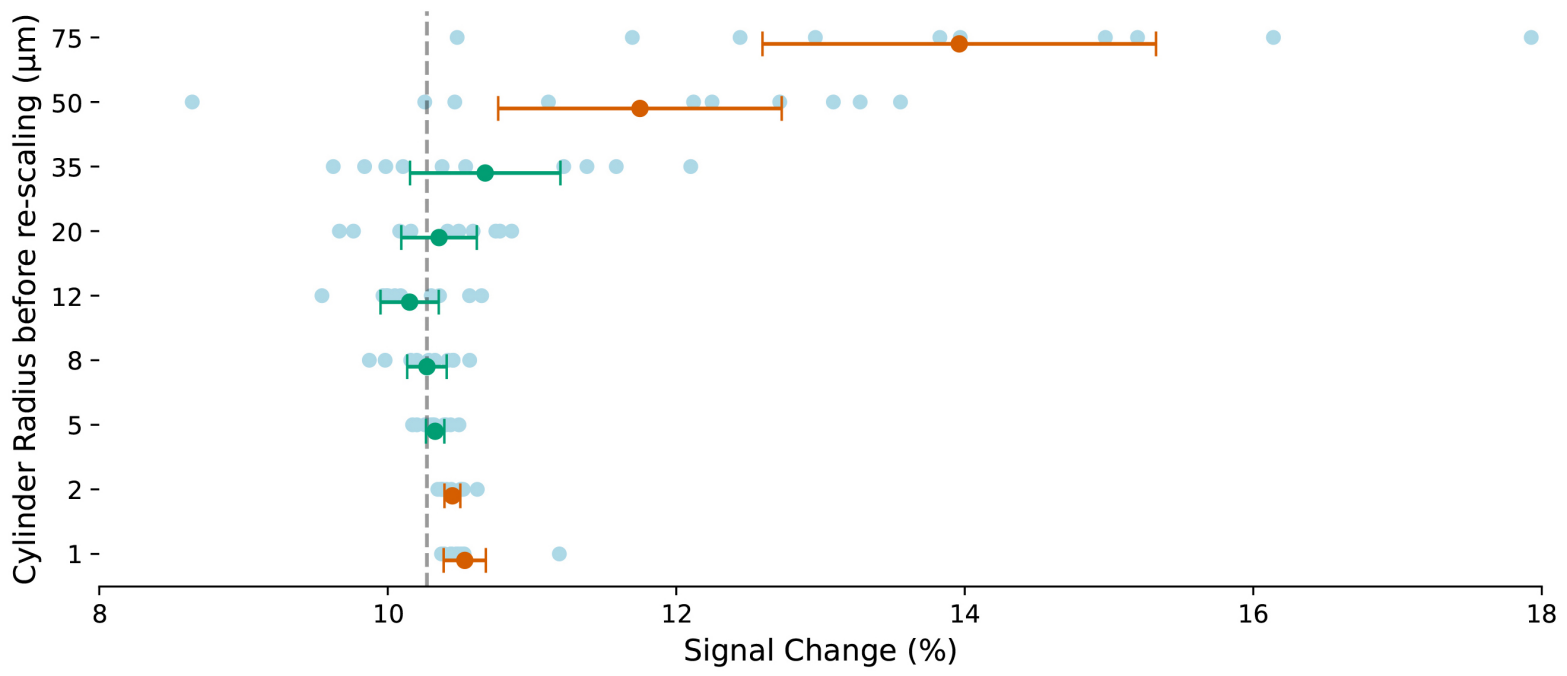
Evaluating accuracy and stability of the FoV scaling approach. Ten phantom with an FoV of 1000 µm and a grid size of 1000 were created, each filled with randomly positioned cylinders of specific sizes. The cylinder sizes were 1, 2, 5, 8, 12, 20, 35, 50, and 75 µm, resulting in a total of 90 phantoms. All phantoms were set with a volume fraction of 4%. Using FoV scaling, these phantoms were adjusted to represent a phantom filled with 8 µm cylinders, and signal changes under GRE sequence were simulated in these environments under both active and rest conditions. The mean and 95% confidence intervals are shown as error bars for each cylinder radius. The average result for phantoms originally containing cylinders with radii of 8 µm was used as the reference mean, indicated by a vertical dashed line.

Figure 1illustrates the accuracy and stability of signal changes when simulating a GRE sequence in phantoms with an FoV of 1 mm, filled with cylinders of various radii and scaled to simulate a phantom filled with 8 µm radius cylinders. The vertical dashed line represents the mean signal change in phantoms originally filled with cylinder of radius 8 µm, serving as the true mean in this evaluation. Error bars denote 95% confidence intervals; they are green if overlapping the true mean and orange if not. For cylinders with initially very large or very small radii, the confidence intervals do not overlap the true mean that was determined from a simulation with a cylinder with a radius of 8 µm. This suggests that such a re-scaling approach may not mimic the behavior of an 8 µm cylinder through the FoV scaling approach in the current form.

**Fig. 2.**
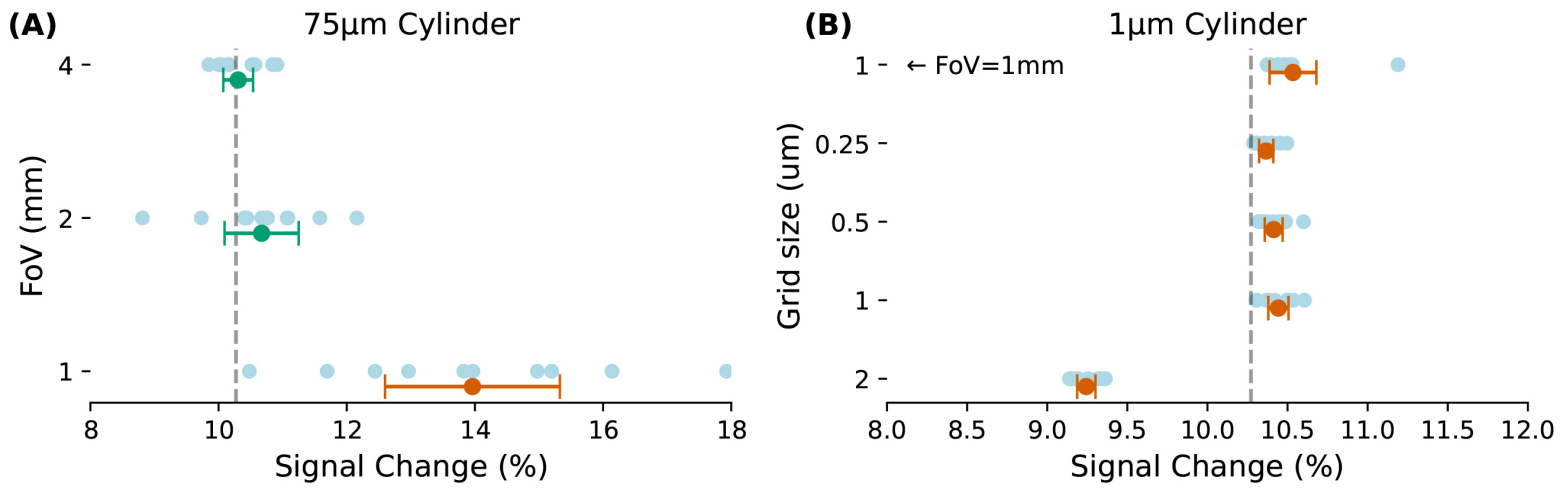
The impact of the grid size and the original FoV on the accuracy and stability of the FoV scaling approach is evaluated. (A) Phantoms with FoVs of 1, 2, and 4 mm were generated, containing randomly positioned cylinders of size 75 µm, with ten phantoms created for each FoV. (B) Phantoms with an FoV of 500 µm were generated with grid sizes of 0.25, 0.5, 1, and 2 µm, each filled with randomly positioned cylinders of size 1 µm, with ten phantoms created for each grid size. Using the FoV scaling approach, these phantoms were scaled to represent a phantom filled with 8 µm cylinders, and the same simulations and analyses as presented inFigure 1were performed. This analysis explores how grid size and FoV affect the convergence of results for small and large cylinders, respectively. In (A), increasing the FoV reduces the confidence interval, bringing it closer to the true mean for large cylinders. In (B), a smaller grid size is observed to reduce the confidence interval, bringing it closer to the true mean for small cylinders. A 1 µm grid size with a 1 mm FoV, as presented inFigure 1, is added for comparison. True mean is taken from the analysis presented inFigure 1.

In the case of a 75 µm cylinder, FoV of the original phantom is expanded from 1000 µm to 2000 µm and 4000 µm. As a result, the confidence interval narrows, and it overlaps with the true mean (Fig. 2A). When the cylinder size is not significantly smaller than the FoV (e.g., 75 µm vs. 1 mm) and the volume fraction is low (e.g., 4%), only a small portion of a cylinder may fit within the phantom. Particles diffusing across the FoV might not experience the full characteristics of that cylinder. Additionally, the random placement of this partial cylinder in the phantom can introduce bias in the results. This effect can be mitigated by using a larger FoV, which may accommodate a complete cylinder rather than just a partial segment, thereby reducing the bias and providing more accurate results.

A similar result is observed when examining phantoms filled with 1 µm cylinders, but with a different adjustment. Here, maintaining a constant FoV but increasing the grid size to achieve smaller voxels in the discrete space tightens the confidence interval and brings it closer to the true mean (Fig. 2B). A phantom with a grid size of 1 µm or larger is not an ideal setup for accommodating cylinders of the same size or smaller, as there are not enough voxels to accurately represent the cylinders and their corresponding off-resonance maps. This issue becomes even more pronounced when the step size is comparable to the cylinder and grid size (in this study, the step size was 0.316 µm for each dimension). Under these conditions, particles can cross vessels in just a few steps, which prevents them from adequately experiencing the dynamics of the local field inhomogeneities created by the presence of the cylinders.

**Fig. 3.**
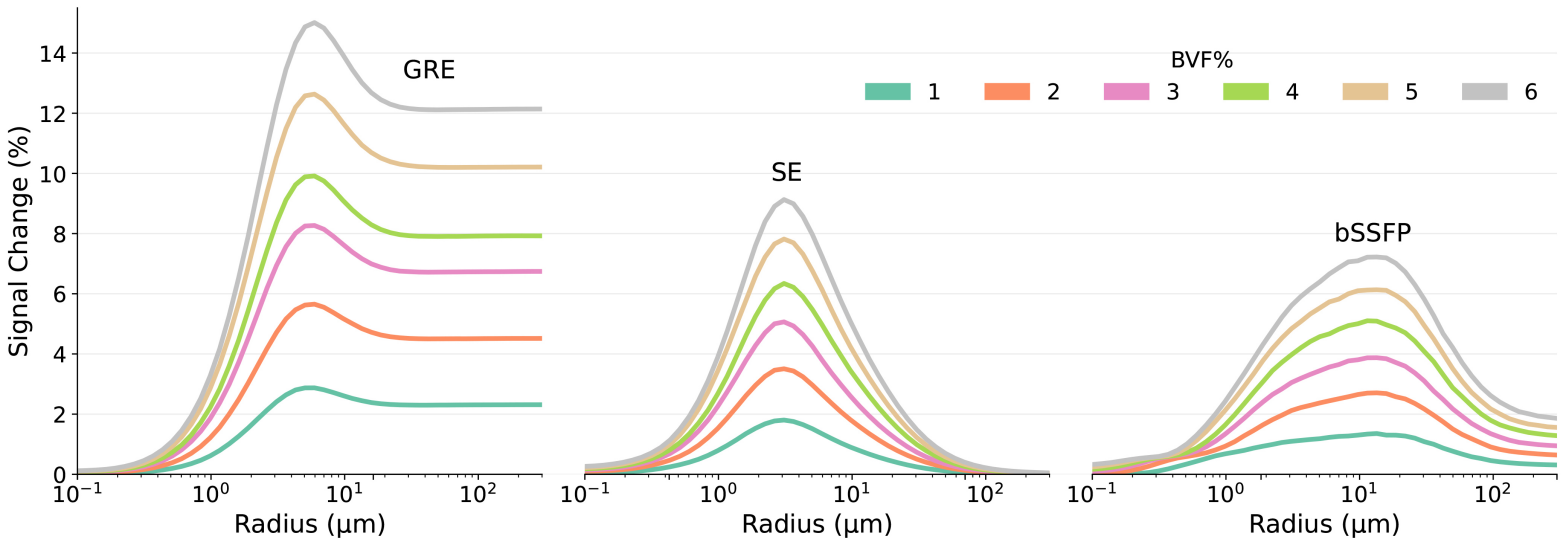
Extravascular BOLD signal change as a function of vessel radius and blood volume fraction was examined under GRE, SE, and bSSFP sequences. Vessels were modeled as cylinders oriented perpendicular to the B_0_direction within a space that has an FoV of 600 µm, a grid size of 1200, and impermeable surfaces. Blood oxygenation levels of 77% for the resting state and 85% for the activated state were assumed. A magnetic field strength of 9.4T is assumed for B_0_.

Figure 3illustrates the BOLD signal change in GRE, SE, and pass-band bSSFP sequences as a function of vessel radius for different BVF values ranging from 1% to 6%. This figure shows that SpinWalk can replicate the results shown inFigure 1ofBáez-Yánez et al. (2017). The sensitivity of the GRE sequence remains consistent for larger vessels and is non-specific for vessels larger than approximately 6 µm. In contrast, the SE sequence shows selective sensitivity, peaking around 4 µm vessels. These findings are in line with previous results of (Báez-Yánez et al., 2017;Boxerman et al., 1995;Weisskoff et al., 1994). While the bSSFP profile is also selective to vessel size, it is broader in comparison to the profile of the SE. The time required to generate data for a single plot (with 50 cylinder radii and two oxygenation levels) was 11.4 ± 2.8 s for GRE, 12.1 ± 3 s for SE, and 2177 ± 125 s for bSSFP. This duration encompasses the total run-time, including all computations, data loading, and result exporting to disk.

**Fig. 4.**
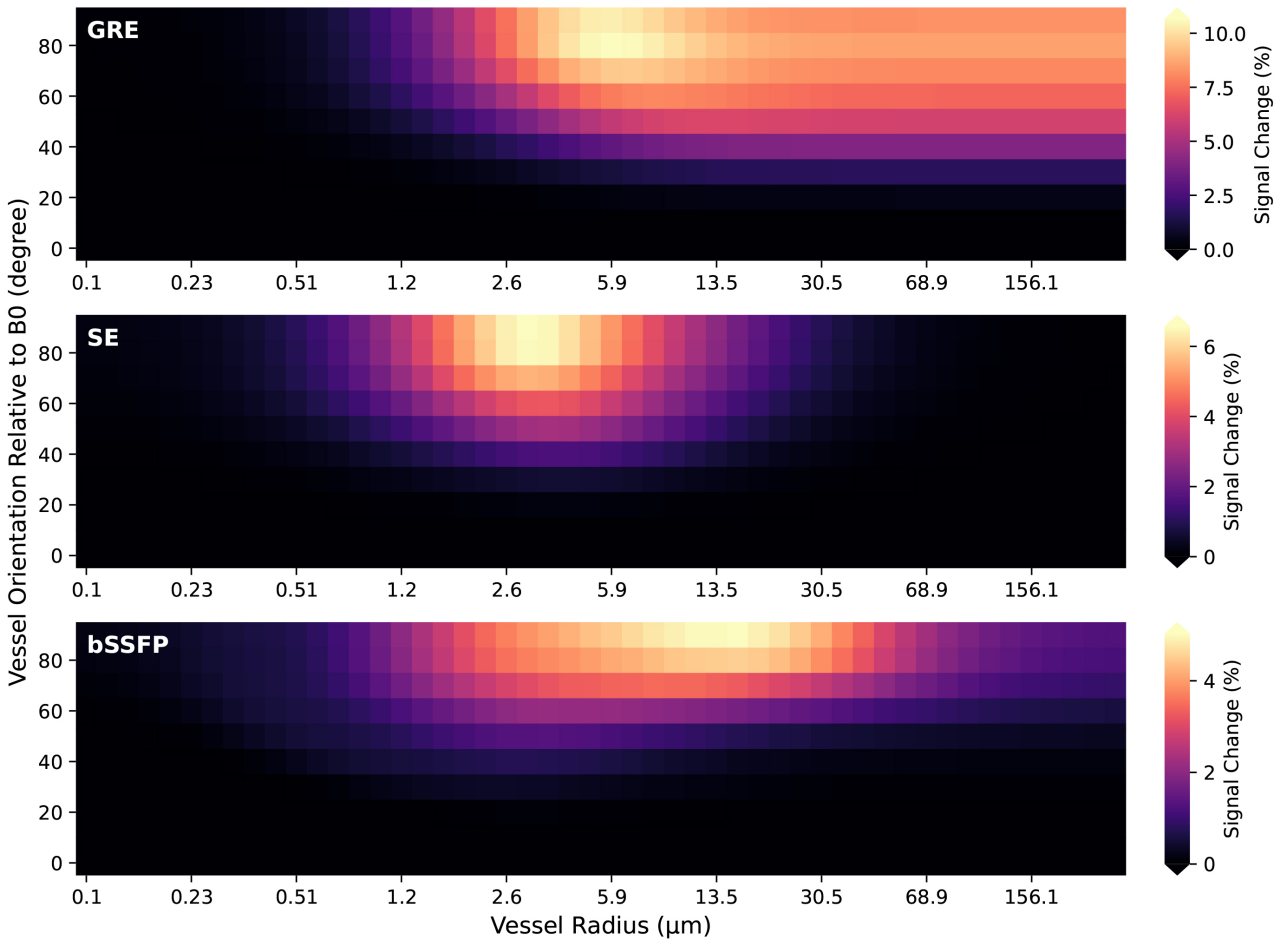
The extravascular BOLD signal change was analyzed as a function of vessel radius and vessel orientation relative to the B_0_(=9.4 T) direction under GRE, SE, and bSSFP sequences. Blood oxygenation levels of 77% for the resting state and 85% for the activated state were assumed. Phantoms with an FoV of 600 µm, a grid size of 1200, and impermeable surfaces were used, with a blood volume fraction of 4%. T1 relaxation times were set to 2200 ms for extravascular and 2500 ms for intravascular compartments. T2 relaxation times were 41 ms for extravascular and 13 ms and 20 ms for intravascular compartments in the resting and activated states, respectively.

Figure 4illustrates the signal changes in GRE, SE, and bSSFP sequences as a function of the orientation of vessels relative to${\vec{B}}_{0}$. All vessels are parallel with respect to each other, and an angle of 0° refers to the vessels being parallel to${\vec{B}}_{0}$. For vessels larger than 1.5 µm in GRE, the signal change is correlated with vessel orientation to${\vec{B}}_{0}$. In SE sequences, this orientation dependence is selective and confined to vessels with radii between 1 and approximately 15 µm. The signal change in bSSFP sequences as a function of vessel orientation is highly dependent on the FA and TR.Figure 4presents only one specific combination (FA = 20°, TR = 10 ms). Under different combinations of FA and TR, bSSFP can be insensitive to vessel orientation or even produce negative BOLD signal changes (not shown) (Scheffler & Ehses, 2016). The simulations of GRE, SE, and bSSFP with SpinWalk for this analysis took 150, 130, and 21500 s, respectively, covering 50 different vessel (cylinder) radii, 10 orientations linearly ranging from 0° to 90°, and two oxygenation levels (rest and active).

**Fig. 5.**
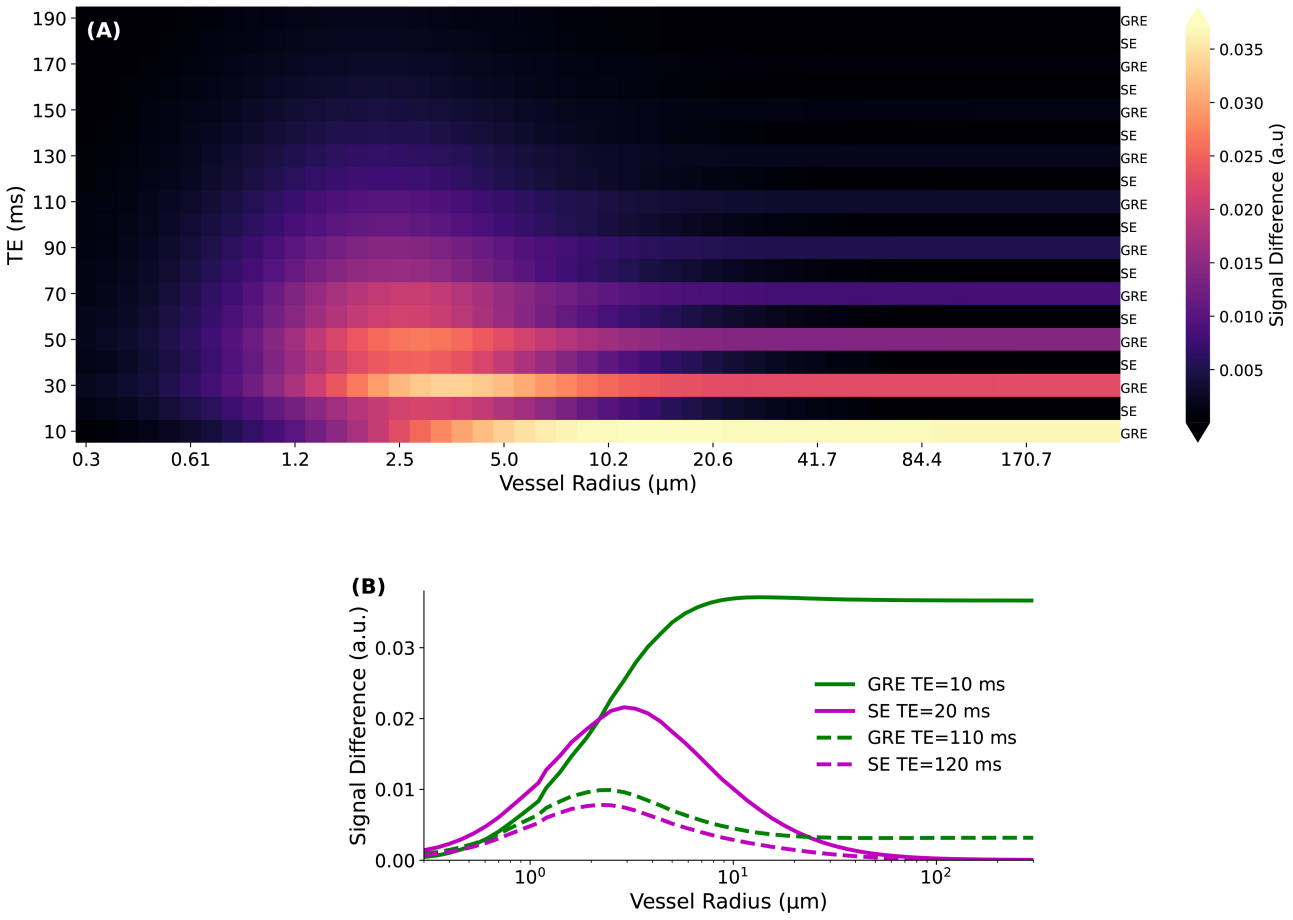
(A) The extravascular BOLD signal change under the GRASE sequence as a function of vessel radius is shown. For consistency withScheffler et al. (2021), the signal change is expressed as the difference between active and rest states and normalized to the number of spins. A total of 19 echoes were collected, with even echoes exhibiting pure SE characteristics and odd echoes displaying a mix of GRE and SE properties. (B) The plots show one even (pure SE) and one odd (mix of GRE and SE) echo acquired at both the start and end of the echo train. Notably, the odd echoes, which resemble GRE, gradually exhibit a hybrid profile combining both GRE and SE characteristics as the echo train progresses.

Figure 5Adepicts the specificity of a GRASE sequence to vessel sizes. This figure replicates the simulation fromFigure 4inScheffler et al. (2021), with minor adjustments regarding the echo spacing. To maintain consistency withScheffler et al. (2021), the signal change is presented as the difference between active and rest states, rather than as a percentage. This difference is normalized to the number of spins. A total of 19 echoes were acquired, with even echoes being pure SE and odd echoes exhibiting a combination of GRE and SE characteristics. Among the odd echoes, the first echo immediately preceding the refocusing pulse is a pure GRE. As we move to subsequent odd echoes, the SE properties become increasingly evident in the profile (Fig. 5B).

**Fig. 6.**
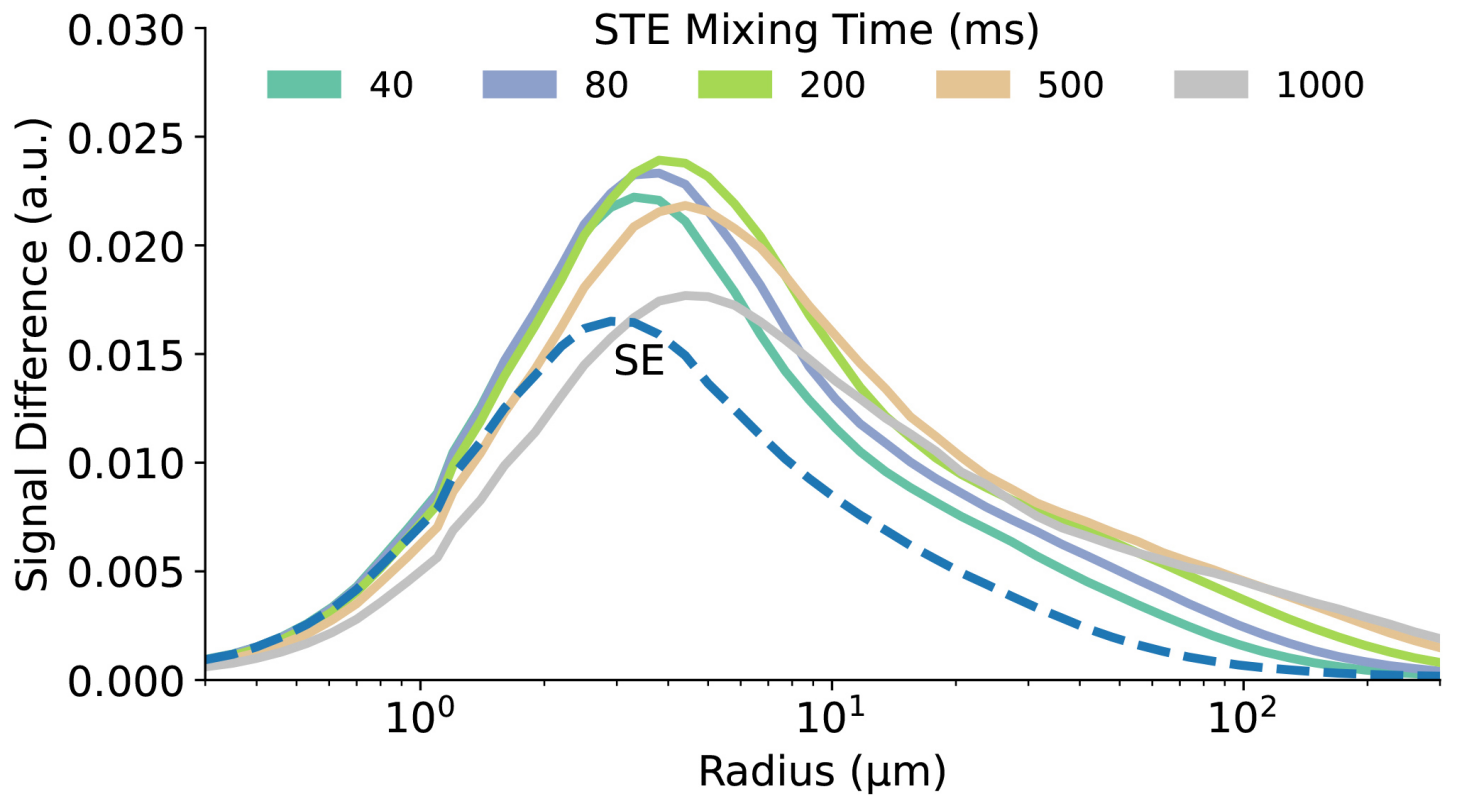
The extravascular BOLD signal changes in the stimulated echo sequence were evaluated as a function of vessel radius and mixing time. The signal change is presented as the difference between active and rest states, rather than as a percentage, to better reflect the T_1_relaxation effect. The results were compared with those from a pure spin echo within the same sequence (dashed line). The initial refocusing RF pulse was applied at 20 ms, generating a spin echo at 40 ms. The mixing time (T_m_, the interval between the refocusing pulses) was individually examined at 40, 80, 200, 500, and 1000 ms.

Figure 6illustrates the effect of mixing time on vessel size specificity in the STE sequence. The primary SE acquired at TE = 40 ms is also shown for reference. The signal change here is also shown as the difference between active and rest states instead of a percentage, to better capture the T_1_relaxation effect. As the mixing time increases, T_1_relaxation becomes more significant, leading to a reduced signal difference between active and rest states. For the examined mixing times, the BOLD signal change is greater than in the SE experiment. When calculated as a percentage, the signal change consistently increases with longer mixing times (results not shown) which is consistent with previously reported simulations and experiments (Goerke & Ugurbil, 2006). The simulation indicates that the peak signal shifts toward larger vessels as the mixing time increases.

**Fig. 7.**
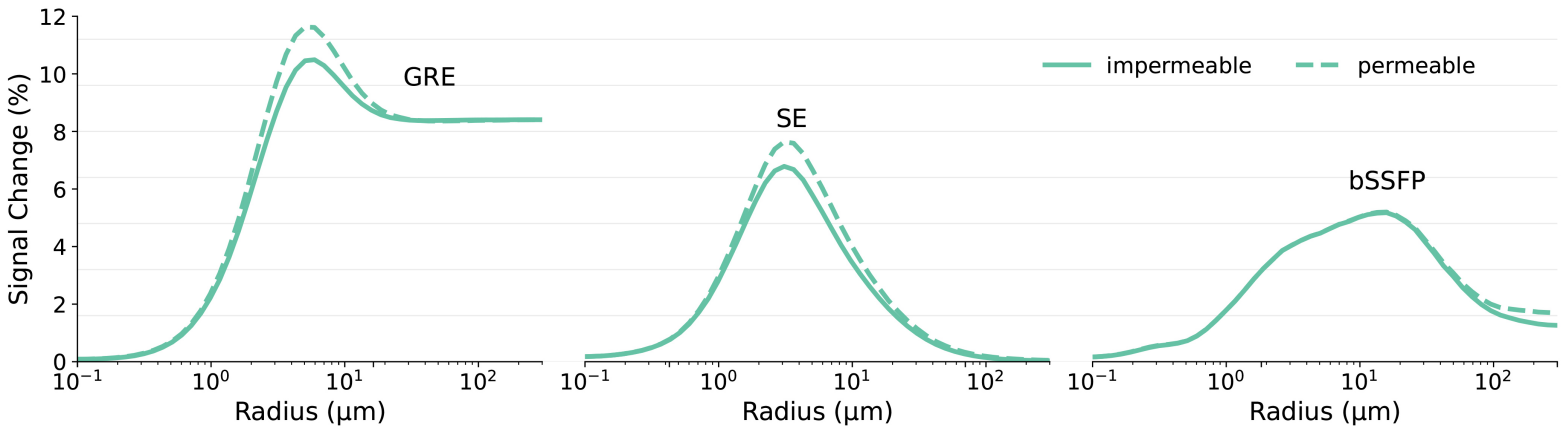
The effect of permeability, with a probability of 10% for spins to transfer from intravascular to extravascular compartments, was examined in GRE, SE, and bSSFP sequences as a function of vessel radius. In this context, spins in small vessels are highly likely to contact the vessel surface and exit the substrate if permeability is present. Blood flow is not considered in the simulation, and spins that exit the vessels are neither replaced nor allowed to return.

Figure 7presents a comparison between impermeable surfaces and 10% permeability from vessel to tissue in GRE, SE, and bSSFP sequences as a function of vessel radius. Here, 10% permeability means that when a particle attempts to move from the intravascular to the extravascular space, there is a probability of 10% that this diffusion will be allowed. It is important to note that in this simulation, the permeability is unidirectional: diffusion from the extravascular to the intravascular space is not permitted. The boundary condition for the FoV that ensures that particles cannot leave the defined simulation area remains enforced throughout the simulations. The simulation indicates that the diffusion of water from the intravascular to the extravascular space can increase the relative signal change in the extravascular space for small vessels in GRE and SE sequences. However, the bSSFP signal behaves differently, with such increases being more pronounced in larger vessels. Particles in smaller vessels are more likely to diffuse into the extravascular space rapidly, causing their magnetization to reach a steady state well before the signal is acquired at echo-time. In contrast, larger vessels retain some particles that gradually diffuse into the extravascular space closer to echo-time. These particles can retain a memory of their prior presence in the intravascular space, influencing the signal characteristics. Note that in this experiment, the spins that diffuse to the extravascular space are neither replaced nor allowed to diffuse back, which impacts the bSSFP results.

**Fig. 8.**
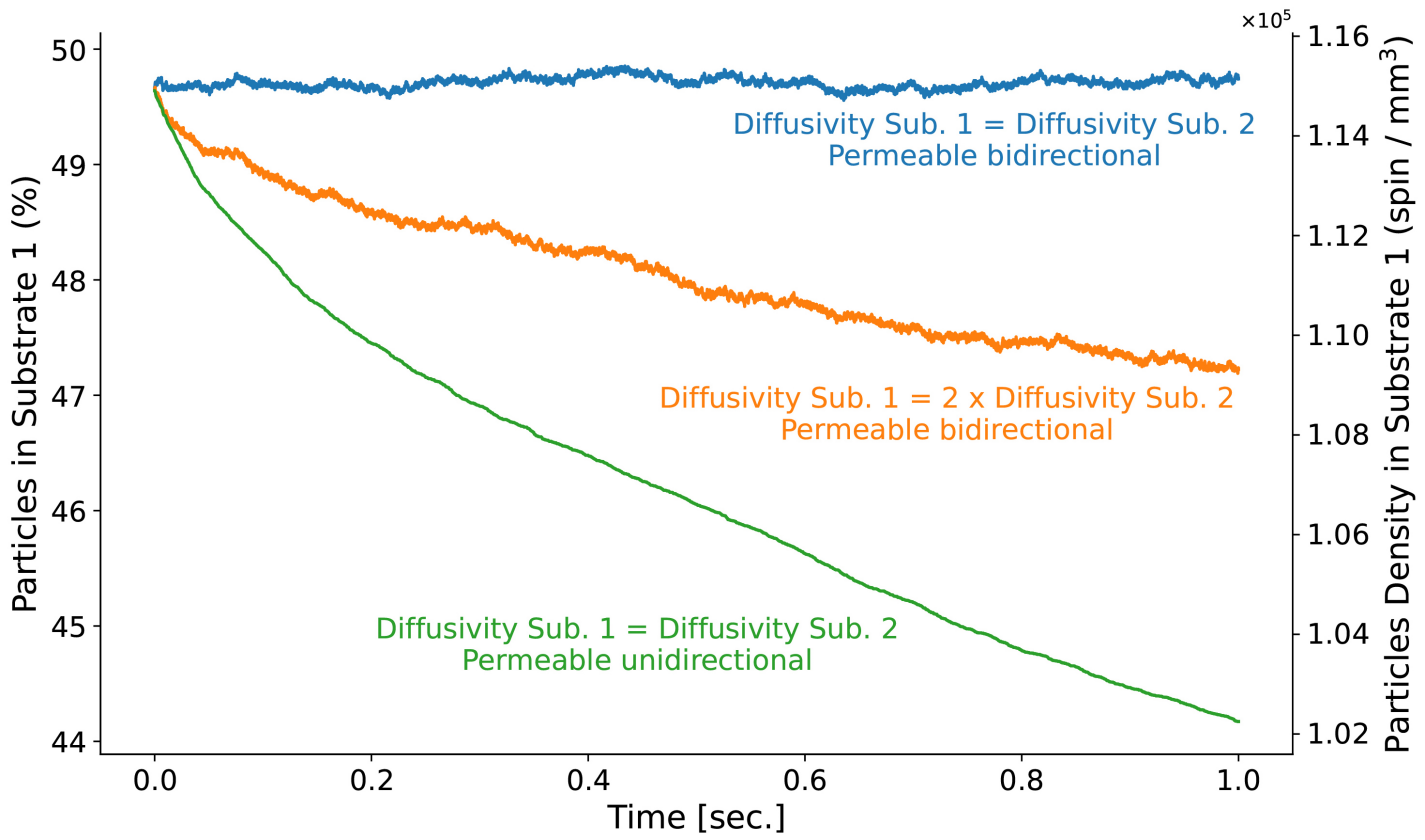
The percentage and density of particles remaining in substrate 1 after 1 s of diffusion were analyzed across three scenarios: 1) both substrates had equal diffusivity and the membrane was bidirectionally permeable; 2) substrate 1 had a higher diffusivity than substrate 2, with the membrane still bidirectionally permeable; and 3) both substrates had identical diffusivity, but the permeability was unidirectional, allowing particles to diffuse from substrate 1 to substrate 2 with 10% probability.

Figure 8illustrates how the equilibrium distribution of spins across the substrates can be influenced by changes in diffusivity or the inclusion of permeability in the simulation. When the two substrates have similar diffusivity and permeability conditions, the spin density shows no significant change throughout the entire simulation. However, when the diffusivity of substrate 1 is doubled, spins gradually accumulate in the low diffusivity regions because the mean residence time is longer. As a result, these low diffusivity regions contribute more to the synthesized signals than their volume fraction would suggest. Finally, the introduction of unidirectional permeability—albeit with a small probability—results in a faster reduction of spins in substrate 1. In just 1 s, more than 10% of the spins in substrate 1 leave and move into substrate 2.

### 5.2 Computing performance

**Fig. 9.**
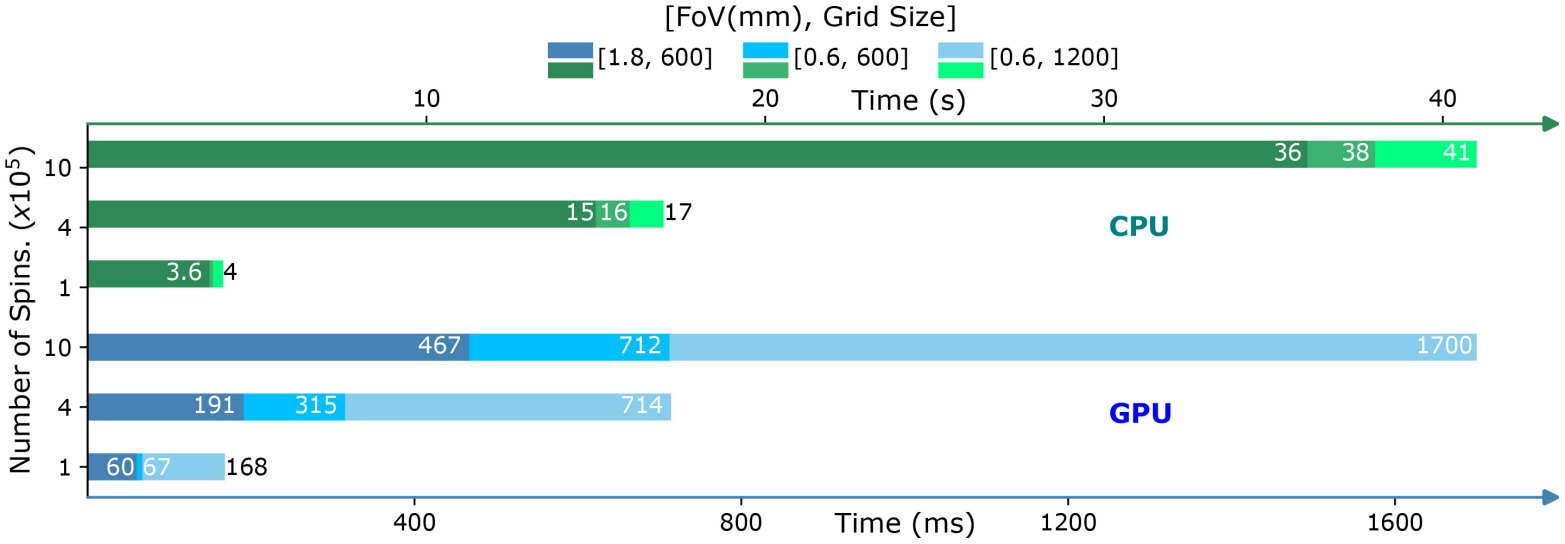
The computing performance of SpinWalk was evaluated on both CPU and GPU with respect to the number of spins, phantom FoV, and phantom grid size. The simulation involved 10^4^random walks of spins in an impermeable environment. The reported elapsed time pertains solely to the simulation itself, excluding the initial overhead associated with loading the phantom and transferring data to DRAM. Notice that the elapsed time is stated in milliseconds for the GPU but in seconds for the CPU for improved visibility.

Figure 9illustrates the performance of SpinWalk when running simulations entirely on a CPU or GPU for 10^5^, 4 x 10^5^, and 10^6^spins. The study reveals that not only do the number of spins and time steps affect the total simulation time, but the FoV and grid size also play a significant role. These parameters dictate the frequency of memory accesses, a critical factor in GPU computations but of less impact in CPU calculations. The bar plot inFigure 9demonstrates that, under the conditions tested and with the hardware used, the same simulation can be executed 23 to 78 times faster on a GPU compared to a CPU. Note that the simulations include off-resonance and relaxation effects; without these, the simulations can be expected to run much faster. Also, the values presented inFigure 9represent the elapsed time for the simulation itself, excluding the initial overhead of loading the phantom and data transfer to DRAM.

The benchmark comparison in the free diffusion experiment among SpinWalk, simDRIFT, and Disimpy shows elapsed times of 1.1, 22, and 35 s, respectively, for the simulation of two b-values. For the simulation of 100 b-values, the recorded elapsed times were 53, 46, and 235 s for SpinWalk, simDRIFT, and disimpy, respectively. The elapsed times for Disimpy and simDRIFT did not scale 50-fold with a 50-fold increase in the number of b-values. This discrepancy is due to the fact that disimpy and simDRIFT are particularly optimized for diffusion simulations. For instance, simDRIFT reuses a single random walk across all b-values, thereby reducing the number of random number generations required. In contrast, SpinWalk generates a new random walk for each b-value.

## 6 Discussion

In this work, we introduced SpinWalk, a Monte Carlo simulator mainly designed for BOLD fMRI applications, but not limited to this application. SpinWalk is a free and open-source software (FOSS) that utilizes either GPU or CPU acceleration, depending on the user’s preference, and can simulate signals of custom MR sequences. It has minimal dependencies, all of which are open source and available for a wide range of hardware architectures and platforms. The capabilities and performance of SpinWalk were exemplarily demonstrated by simulating various sequences in different scenarios and successfully replicating results from previous studies.

To evaluate BOLD signal change across different vessel sizes, the simulations in this work employed an FoV scaling approach. This method allows for the simulation of a wide range of vessel sizes without the need to create numerous numerical phantoms. FoV scaling ensures a constant BVF across simulations and maintains consistent cylinder placement, leading to more uniform simulations. However, careful selection of the original unscaled phantom is crucial, taking into account the range of downscaling and upscaling, as well as the ratio of vessel size to FoV and grid size in the original unscaled phantom. The results obtained from the FoV scaling analysis (seeSection 5) for very small and large cylinder radii prompted a deeper investigation into the factors that may influence stability and accuracy. This additional analysis aimed to identify and understand the variables that significantly impact the reliability of the simulations. These limitations were characterized and described in detail. It is also important to note that the FoV will not remain identical for different vessel sizes after scaling. Conversely, placing a large vessel within a small FoV while maintaining a small BVF can be challenging, making FoV scaling particularly useful in such cases. This approach, of course, is limited to the case of spatially isotropic cylinders as already mentioned. In case of more sophisticated models that, for instance, mimic pial and penetrating vessels, there is most likely not much sense in scaling the entire model.

### 6.1 Further performance improvements

One aim in developing SpinWalk was to reduce the runtime of simulating steady-state MR sequences like bSSFP. SpinWalk now enables such simulations to be completed in just a few minutes rather than in several hours, not only for analytically described vascular architectures but also for arbitrary geometries. However, profiling analysis and insights from related literature reveal that there is still potential for performance improvement. Notably, generating random numbers for the random walk and memory bandwidth were identified as performance bottlenecks. It has been noted that the time needed to transfer data between RAM and DRAM in numerous simulations is often as long as, or even longer than, the time required for the simulation process itself. Furthermore, each 3D random walk involves three random number generations, and additional random walks may be required if the simulated trajectory exits the region defined by the substrate (impermeability) or the restricted environment (FoV).

To address these issues, random walks can be conducted in polar coordinates with fixed step sizes, reducing random number generation to just two per time step (Lee et al., 2021;Rafael-Patino et al., 2020). Alternatively, random walks can be further optimized by using precomputed look-up tables for azimuthal and polar angles, reducing random number generation to only one per time step (Sandgaard et al., 2024). As shown inFigure 9, increasing voxel mesh resolution leads to slower calculations due to limited memory bandwidth, which must support the demands of thousands of CUDA cores simultaneously. In contrast, CPU calculations are less affected by this issue because they involve a lower degree of parallelism. Many Monte Carlo simulators use triangular meshes as input to define substrate surfaces, which are relatively memory-efficient and helpful here but can be computationally intensive (Riedmatten et al., 2024). CACTUS (Villarreal-Haro et al., 2023) and ConFiG (R. Callaghan et al., 2020) utilize triangulated meshes to represent substrate geometry, which can be computationally demanding. In contrast, MEDUSA (Ginsburger et al., 2019) breaks down each microstructural element into a set of overlapping spheres, offering greater efficiency in terms of both memory usage and computational requirements. However, it is important to consider how to deal with off-resonance effects in 3D space when using such meshes. To calculate the field inhomogeneity in complex geometries, the finite perturber method is preferred for its simplicity of implementation (Pathak et al., 2008), which requires a voxel representation of the substrates. Voxelization methods, such as the signed distance function or ray-casting base (Roth, 1982;Winther et al., 2024) method, can be utilized to convert surface mesh representations—like those produced by CACTUS and ConFiG—into a voxel representation.

There is a trade-off between computational performance and the versatility of the simulator. For narrowly defined targets with specific assumptions, performance can be highly optimized, as demonstrated with simDRIFT. In contrast, SpinWalk accounts for a range of factors, including off-resonance effects, relaxation, and permeability, and allows for a variable number of RF pulses and gradients. This flexibility enables the simulation of numerous sequences and evaluates a wide array of parameters.

### 6.2 Limitations and exclusions

Most numerical simulations typically depend on several assumptions regarding tissue models and pulse sequences. SpinWalk follows some of this approach to achieve performance gains too. In each time step, sequence components are applied sequentially: first, a random walk is performed; then, ideal dephasing is applied, followed by the application of gradients, RF pulses, and finally, the recording of the echo and trajectory. Thus, RF and gradients are not applied simultaneously.

In the simulation of permeability, spins exiting the substrate are not compensated. This must be carefully considered when analyzing long-duration sequences, as no spins may remain within the substrate (e.g., in intravascular space). This bias is particularly relevant for simulations conducted over relatively long durations. This phenomenon is observed in the bSSFP sequence results depicted inFigure 7. To prevent a reduction in SNR, it is crucial to maintain a constant average spin density. If spin density is not preserved, the effect may be further amplified in cases of varying diffusivity between two adjacent permeable substrates, where spins tend to accumulate in regions with lower diffusivity (Fig. 8), potentially introducing bias into simulations (Fieremans & Lee, 2018). This bias is typically negligible in short-duration simulations like GRE and SE but can be significant in bSSFP. In the simulations presented in this study, both intravascular and extravascular regions were modeled with identical diffusivity. In in vivo experiments, fresh spins introduced by flow are expected, leading to increased signal changes in bSSFP, particularly in small vessels. Addressing constant permeability and maintaining spin density are potential areas for improving the simulations.

While SpinWalk can generate numerical phantoms filled with cylinders or spheres for demonstration of simple models, it is not capable of generating vascular anatomical network (VAN) or realistic cell models. These models, as well as the resulting off-resonance maps, need to be generated separately and fed to SpinWalk, as the primary aim of the SpinWalk is to facilitate versatile sequence simulation while being high performance.

Phantoms with voxel mesh representation are relatively large. One should consider the DRAM capacity of their GPU, as the entire phantom data will be copied there. Nowadays, many modern GPUs are equipped with at least 16 GB of DRAM, which should be sufficient for a grid size of 1400 in each dimension. This corresponds to a voxel mesh resolution of 0.5 µm for an FoV of 700 µm. For a higher voxel resolution, FoV should be reduced. Theoretically, in case limitations are faced at this point, one could consider splitting up the matrix into several sub-matrices that fit the available memory. This would inevitably result in an additional overhead of accessing memory and reduce the performance, but, in principle, would allow arbitrary matrix sizes to be used.

## Data Availability

The source codes for SpinWalk are available for download from its GitHub repository (https://github.com/aghaeifar/SpinWalk). The scripts to reproduce the results presented in this work are available athttps://github.com/aghaeifar-publications/SpinWalk_demo. The simulations in this work were conducted using SpinWalk version 1.15.
